# Supplementary material for: Bat Accelerated Regions Identify a Bat Forelimb Specific Enhancer in the HoxD Locus
Source: PLoS Genet. 2016 Mar 28;12(3):e1005738. doi: 10.1371/journal.pgen.1005738 (PMC4809552; doi:10.1371/journal.pgen.1005738)
Supplement: S1 Fig — Insets showing higher magnification images of all embryos that had limb LacZ staining are shown next to the whole embryo. (PDF) [file pgen.1005738.s001.pdf]

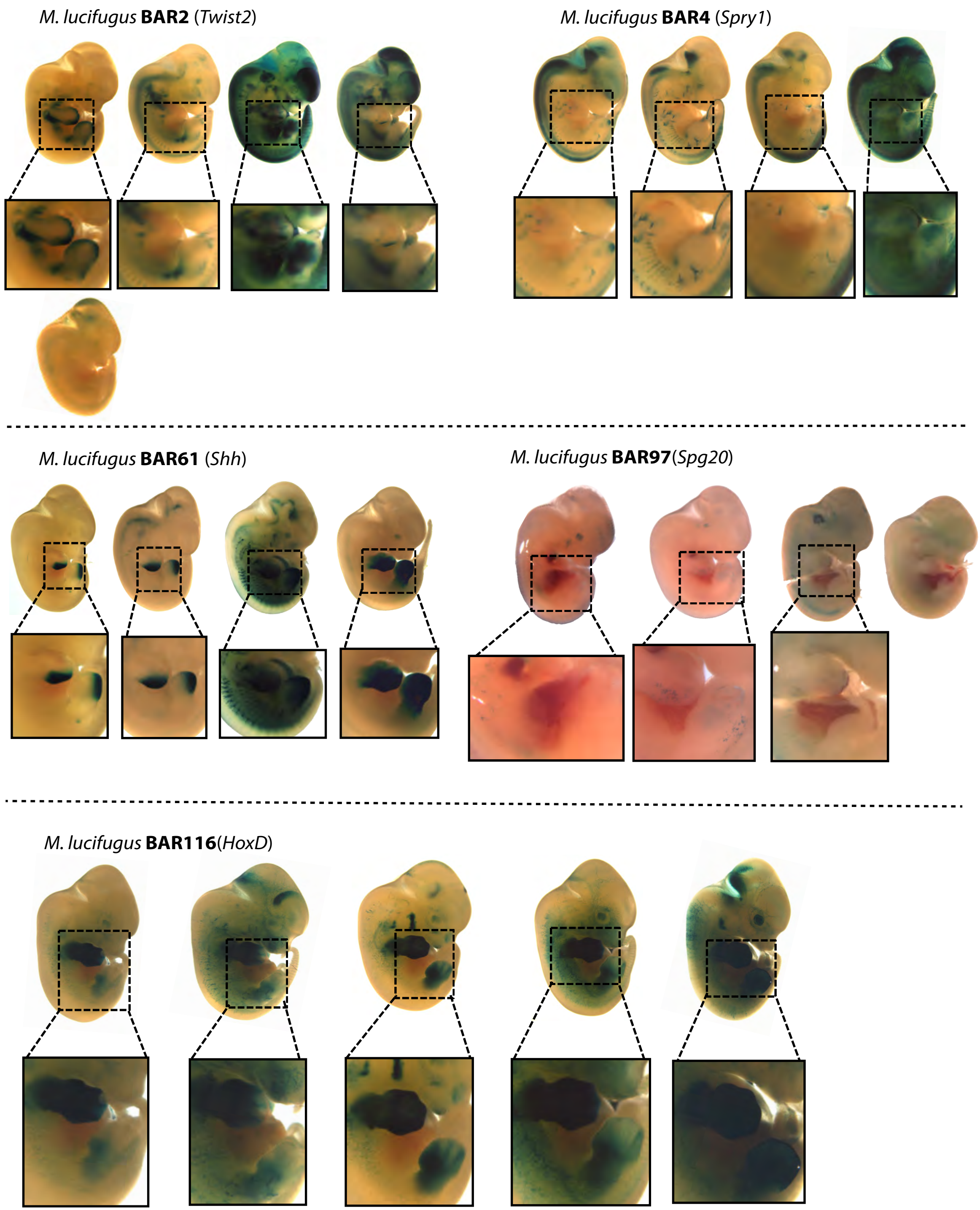

M. lucifugus **BAR2** (*Twist2*)
M. lucifugus **BAR4** (*Spry1*)
M. lucifugus **BAR61** (*Shh*)
M. lucifugus **BAR97**(*Spg20*)
M. lucifugus **BAR116**(*HoxD*)
