# Supplementary figures and images for: Bat Accelerated Regions Identify a Bat Forelimb Specific Enhancer in the HoxD Locus
Source: PLoS Genet. 2016 Mar 28;12(3):e1005738. doi: 10.1371/journal.pgen.1005738 (PMC4809552; doi:10.1371/journal.pgen.1005738)

mouse **BAR4** (*Spry1*)

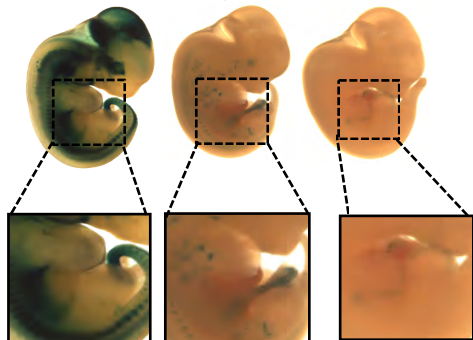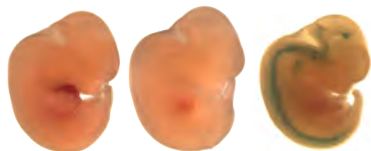

mouse **BAR61** (*Shh*)

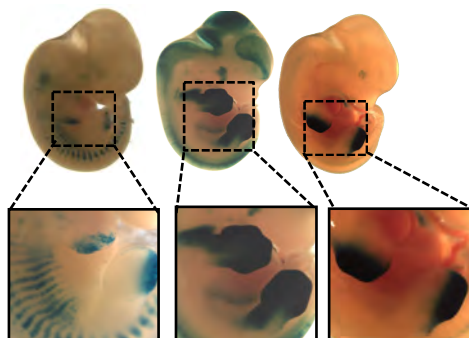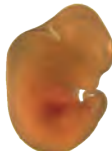

mouse **BAR97** (*Spg20*)

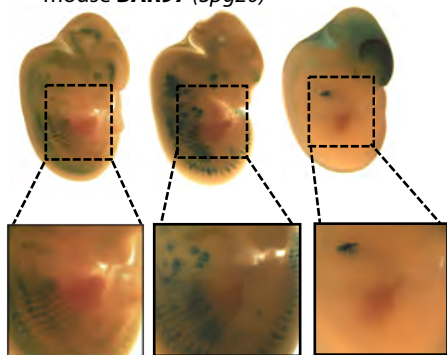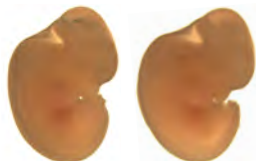

mouse **BAR116** (*HoxD*)

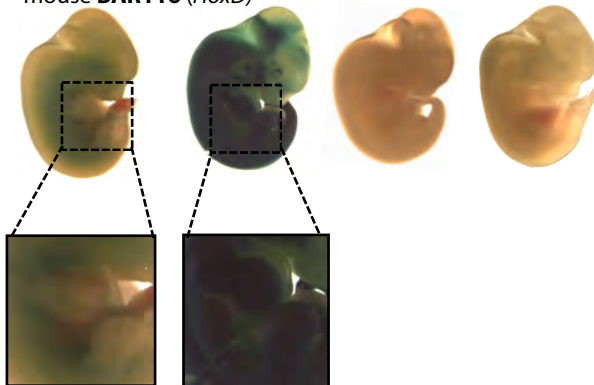

Combined **BAR116** (*HoxD*)

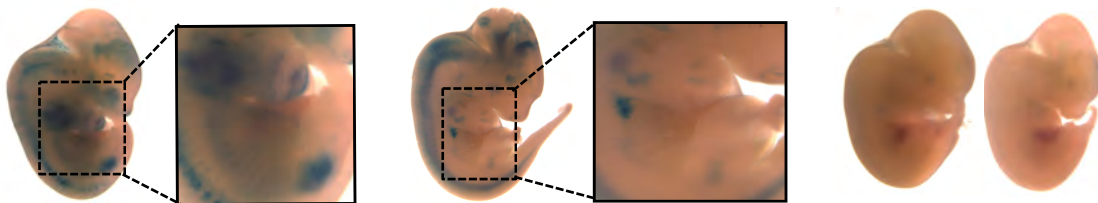

Supplement: S2 Fig — Insets showing higher magnification images of all embryos that had limb LacZ staining are shown next to the whole embryo. (PDF) [file pgen.1005738.s002.pdf]
