# Supplementary material for: Bat Accelerated Regions Identify a Bat Forelimb Specific Enhancer in the HoxD Locus
Source: PLoS Genet. 2016 Mar 28;12(3):e1005738. doi: 10.1371/journal.pgen.1005738 (PMC4809552; doi:10.1371/journal.pgen.1005738)
Supplement: S3 Table — Bat specific in situ hybridization probes were generated using primers given. Here, we provide the amplicon lengths and GenBank identification numbers for Bat Hoxd10-13 WISH probe sequences. (DOCX) [file pgen.1005738.s005.docx]

### Table S3: Bat probe primers and GenBank numbers for the amplified sequences.

| Primer Name | Primer Sequence (5’- 3’) | nt | GC (%) | Tm  (°C) | Amplicon size (bp)  GENBANK ID |
| --- | --- | --- | --- | --- | --- |
| ISH_bHoxd10_Fwd | CCAACAGCTCTCCTGCTGCTAATACT | 26 | 50 | 67.2 | 920 AAPE02004649.1 |
| ISH_bHoxd10_Rvs | GACCTGCCTGTCGGTGAGGTTA | 22 | 59 | 67.2 |  |
| ISH_bHoxd11_Fwd | TCGGACTTCGCCAGCAAGCCGTC | 23 | 65 | 72.5 | 736  AAPE02004650.1 |
| ISH_bHoxd11_Rvs | GCCGGTCAGTGAGGTTGAGCATCCGAG | 27 | 63 | 73.1 |  |
| ISH_bHoxd12_Fwd | ACTCCTTCTACTTCTCCAACCTGCG | 25 | 52 | 66.8 | 645  AAPE02004650.1 |
| ISH_bHoxd12_Rvs | TTGGACAATTCCTTGCGCTTCTGC | 24 | 50 | 67.5 |  |
| ISH_bHoxd13_Fwd(iii) | ACGGCTACCACTTCGGCAACG | 21 | 62 | 68.8 | 501  AAPE02018005.1 |
| ISH_bHoxd13_Rvs(i) | CGTTCTCCAGTTCTTTGAGC | 20 | 50 | 60.2 |  |

### Bat specific ISH probes were generated using primers given. Here, we provide the GENBANK IDs for Bat Hoxd10-13 WISH Probe Sequence.
